# Recombinant Zika virus envelope protein elicited protective immunity against Zika virus in immunocompetent mice

**DOI:** 10.1371/journal.pone.0194860

**Published:** 2018-03-28

**Authors:** Huabin Liang, Ruoheng Yang, Zhihua Liu, Min Li, Haitao Liu, Xia Jin

**Affiliations:** 1 Viral Disease and Vaccine Translational Research Unit, CAS Key Laboratory of Molecular Virology and Immunology, Institut Pasteur of Shanghai, Chinese Academy of Sciences, Shanghai, China; 2 University of Chinese Academy of Sciences, Beijing, China; Instituto Butantan, BRAZIL

## Abstract

Zika virus (ZIKV) has caused great public concerns due to its recent large outbreaks and a close association with microcephaly in fetus and Guillain-Barre syndrome in adults. Rapid development of vaccines against ZIKV is a public health priority. To this end, we have constructed and purified recombinant ZIKV envelope protein using both prokaryotic and eukaryotic expression systems, and then tested their immunogenicity and protective efficacy in immune competent mice. Both protein immunogens elicited humoral and cellular immune responses, and protected immune competent mice from ZIKV challenge *in vivo*. These products could be further evaluated either as stand-alone vaccine candidate, or used in a prime-and-boost regimen with other forms of ZIKV vaccine.

## Introduction

Zika virus (ZIKV) is an emerging mosquito-transmitted flavivirus that belongs to the *Flaviviridae* family. It contains a single-stranded, positive-sense RNA genome [1]. ZIKV was first isolated from a rhesus macaque in 1947, but it has caused very few human infections for six decades. Most human infections are manifested as a self-limited febrile illness that presented as low-grade fever, rash, headache, conjunctivitis, myalgia and arthralgia [2–4]. But the unprecedented large Zika outbreaks in French Polynesia and South America more recently have revealed an association between ZIKV infection and the increased incidence of microcephaly in fetus and infants and Guillain-Barré syndrome in adults [5–7]. Additionally, the unusual properties of sexual and vertical transmission, and prolonged presence of infectious virus or viral RNA in a variety of body fluids including tears, saliva, urine and semen, further complicated the prevention and control of ZIKV infection [8–13]. Although the underlying mechanisms of rapid dissemination of ZIKV in certain countries and regions are still being investigated [14], it has been speculated that ZIKV might pose a long-term international threat that requires substantial attention [15], including the rapid development of a preventative vaccine.

Several approaches have been attempted towards the development of a Zika vaccine. These include traditional purified inactivated virions, live attenuated viral vaccines, recombinant subunit vaccines, gene-based vaccine platforms (DNA or mRNA), an adenovirus vectored vaccine, and virus like particles (VLP). These vaccines have been demonstrated varible efficacy in mice and nonhuman primate models [16–24]. Among them, two DNA vaccines have finished Phase I human clinical trials [25]. Similar to all preclinically tested vaccines, the final vaccine product to be marketed will be dertemined by efficacy testing in Phase III clinical trial.

Recombinant subunit-based vaccine strategy is an alternative platform that warrants testing because of its remarkable safety, relative ease for antigen production, and modest cost. The choice of antigen is straightforward. Similar to other flaviviruses, the ZIKV RNA genome encodes one open reading frame (ORF) that is posttrasnaltionally processed into three structural proteins (C, capsid; prM, pre-membrane; E, envelope) and seven non-structural proteins (NS1, NS2A, NS2B, NS3, NS4A, NS4B and NS5). The outer surface of mature ZIKV virion consists of 180 copies of E glycoproteins, which is arranged into 90 antiparallel homodimers and anchored onto the virus lipid envelope via a transmembrane domain [26, 27]. The N-terminal ectodomain of an individual E glycoprotein (approximate 80% length of the entire E protein, herein as E80) is comprised of three distinct antigenic and structural regions (EDI, EDII and EDIII) that mediate viral attachment and post-entry endosomal fusion during the virus entry process [28]. Thus, E protein is an obvious antigen of choice. Furthermore, epitope mapping analysis in mouse models has suggested potential roles of E protein in inducing protective T cell and B cell responses [29, 30], highlighting the importance of E protein for Zika vaccine development.

In this study, we constructed E protein-based subunit vaccines (ZIKV E80) in both *E*.*coli* and *Drosophila* expression systems, and examined the immunogenicity and protective efficacy of these two ZIKV E80 proteins. We found that both candidate vaccine products generated neutralizing antibodies and antigen-specific T cell responses. Importantly, these candidate vaccines conferred protection against ZIKV challenge in immunocompetent mice.

## Materials and methods

### Ethics statement

All animal experiments were performed following the guidelines of care and use of laboratory animals by the Ministry of Science and Technology of the People's Republic of China and approved by Institutional Animal Care and Use Committee of the Institut Pasteur of Shanghai (IPS), Chinese Academy of Sciences. All mice were bred in specific pathogen free (SPF) individually ventilated cages (IVC) and were given standardized diets at the Institut Pasteur of Shanghai animal facilities. After each experiment, the mice were euthanized by CO_2_ asphyxiation. ZIKV infection experiments were performed in biosafety level 2 (BSL-2) and animal biosafety level-2 (ABSL-2) containments with the approval of Institutional Biosafety Committee at IPS.

### Cells and viruses

Vero cells (African Green monkey kidney epithelial cells) (ATCC) were passaged in Dulbecco’s modified Eagle’s medium (DMEM, Gibco) plus 10% heat-inactivated fetal bovine serum (FBS, Gibco) and 1% penicillin/streptomycin (P/S, Invitrogen) at 37°C in a 5% CO_2_ incubator. Mosquito C6/36 cells (ATCC) were cultured in Minimum Essential Medium (MEM, Gibco) supplemented with 10% FBS, 1% P/S, 1% non-essential amino acids (NEAA, Gibco) at 28°C and 5% CO_2_. Drosophila S2 cells (Invitrogen) were cultured at 28°C in Express Five Serum free medium (SFM, Gibco) supplemented with 1% glutamine and 1% P/S or Schneider’s Drosophila medium (SDM, Gibco) supplemented with 10% FBS and 1% P/S. *Escherichia coli* BL21 and DH5α competent cells were cultured in LB medium at 37°C in spinner glass at the speed of 220 rpm/minute. For plate culture, 15% agar was added to LB medium. Kanamycin was added at a concentration of 50 μg/ml for positive colony selection.

Zika virus (ZIKV, SZ-WIV01 strain) was obtained from the Wuhan Institute of Virology of Chinese Academy of Sciences and propagated in mosquito C6/36 cells at a multiplicity of infection (MOI) of 0.05. ZIKV stocks were titrated on Vero cell monolayer, and stored at -80°C until use.

### Plasmids construction and protein expression

The gene encoding 80% N-terminal of Zika virus (ZIKV, SPH2015 strain) envelope protein (E80, amino acid residues 1 to 408) was optimized for expression, synthesized and cloned into the pUC57 plasmid (GenScript, Nanjing, China). The E80 sequence was amplified from recombinant plasmids and then cloned into either pMT/Bip/V5-His A plasmid (Invitrogen) using XbaI and Bgl II restriction sites (pMT/Bip/V5 His A-ZIKV E80 plasmid), or pET30a plasmid with Nde I and Hind III cloning sites (pET30a-ZIKV E80 plasmid). The recombinant pMT/Bip/V5-His A-ZIKV E80 plasmid was co-transfected with a pCoblast selection plasmid which contains a blasticidin resistance gene into *Drosophila* S2 cells using a Calcium Phosphate Transfection kit (Invitrogen). The transfected S2 cells were selected by blasticidin (25μg/ml) for four weeks in SDM, and then the survived cells were used for the production of recombinant ZIKV E80 protein (S2 ZIKV E80, E80_S) with 10μM CdCl_2_ induction in SFM with 10μg/ml blasticidin. Cell supernatant was collected and concentrated with 3kD centrifugal filter tubes (Millipore) for further purification of native proteins using Ni-NTA beads. The purified E80_S protein was dialyzed in PBS, which was changed 3 times for a complete cycle of dialysis. For prokaryotic expression of the E80 protein, the BL21 Codon plus *Escherichia coli* was transformed pET30a-ZIKV E80 plasmid and the recombinant ZIKV E80 protein (*E*.*coli* ZIKV E80, E80_E) was expressed by IPTG induction. Under this condition, most of the E80_E protein existed within the inclusion body. Thus, we collected the inclusion bodies by ultrasonication and centrifugation and then dissolved them using 6M guanidine hydrochloride buffer. The Ni^2+^ affinity chromatography-based purification was then performed under denaturing condition according to the manual provided by the Ni-NTA agarose supplier (Thermo Fisher Scientific). The purified E80_E protein was then renatured by adding various concentration of Urea buffer (8M-6M-4M-2M-0M). Both forms of recombinant protein were sterilized by 0.22μm MILLEX® GP filter unit (Merck Millipore) and stored in -80°C.

### SDS-PAGE and Western blot

ZIKV E80 proteins expressed were verified by SDS-PAGE and Western blot analyses. Briefly, purified ZIKV E80 proteins were diluted in SDS-PAGE sample loading buffer and boiled at 100°C for 10 minutes and then separated on a 10% SDS-PAGE gel. Gel was either stained with Coomassie brilliant blue G-250 (Thermo Scientific) or transferred onto a polyvinylidene difluoride (PVDF) membrane (Millipore). The membrane was probed with an anti-His tag monoclonal antibody (Proteintech) or an anti-ZIKV envelope monoclonal antibody (4G2, Biofront technology), followed by a secondary anti-mouse IgG antibody (Promega) and finally colorized using the BCIP/NBT Alkaline Phosphatase Color Development Kit (Beyotime).

### Immunization

Specific-pathogen-free (SPF), 6–8 weeks old female BALB/c mice were purchased from Beijing Vital River Laboratory Animal Technology (licensed from Charles River). E80_S or E80_E proteins were formulated with alum adjuvant (Alhydrogen, InvivoGen) at 10μg or 50μg doses, using PBS formulated in alum as a negative control, and then administered subcutaneous at the base of tail to 5–6 mice per group at weeks 0, 2 and 4. Blood or spleen samples were obtained at various time points for detecting antibody and T cell responses. Blood samples from each group of mice were collected at two weeks after the third immunization. Serum samples were collected by centrifugation and kept at -80°C until use.

### *In vivo* protection assay

To examine the *in vivo* protection conferred by vaccination in immune competent wild-type mice, we adapted a recently developed model that transiently blocks type I interferon activity by antibody to interferon receptors (IFNR) [17, 31–34]. Briefly, immunized mice were injected with 2mg anti-IFNR Abs (BioXcell) intraperitoneally (i.p) one day prior to challenge with 1*10^5^ PFU ZIKV (SZ-WIV01) by subcutaneous (s.c) injection. For adoptive transfer, 300μl heat-inactivated immune sera or control sera (a mixture from different mice of the same experimental group) was administered i.p. to each recipient mouse at 4 hours before infection. Blood was extracted by retro-orbital bleeding starting at 1 day post infection (DPI) for 5 consecutive days, and then centrifuged to obtain sera. To quantify virus infectivity, 10μl serum sample from each infected mouse was inoculated onto Vero cells. After being cultured at 37°C for 4 days in DMEM containing 1% FBS, cells were harvested. The percentage of infected cells was determined by staining with an anti-E mouse monoclonal antibody (4G2), followed by staining with an anti-mouse AF488 antibody (Invitrogen), and then analyzed by flow cytometry (FACS). Weight of each mouse was also recorded from 1 to 14 DPI to evaluate the protective efficacy of recombinant ZIKV E proteins.

### Enzyme-linked immune sorbent assay

Enzyme-linked immune sorbent assay (ELISA) was used to determine the antibody response levels elicited by *E*.*coli* and S2 expressed ZIKV E80 proteins using serum samples obtained from immunized mice, and the cross reactivity of ZIKV immune sera with DENV E80 protein. Briefly, 96-well flat-bottom plates (Costar) were coated overnight with 100μl of diluted ZIKV E80 protein or DENV-3 E80 protein, at a final concentration of 0.5μg/ml. The plates were decanted and then blocked with 5% no-fat milk in PBS containing 0.05% Tween 20 (PBST) for 2 hours at 37°C. Two fold diluted inactivated serum samples were then added in duplicate wells and incubated for 1 hour at 37°C. Horseradish peroxidase (HRP)-conjugated anti-human antibody (Invitrogen) was then added and incubated for 1 hour. Color development was performed using TMB single solution (Life technology), followed by the colorimetric analysis at 450 nm in a 96-well plate reader (Thermo Fisher Scientific).

### Plaque reduction neutralization test

To characterize the levels of anti-ZIKV neutralizing antibodies in mice, the plaque reduction neutralization test (PRNT) was preformed and 50% neutralization serum concentration (PRNT_50_) was calculated using Probit analysis (SPSS, 19th version). Briefly, Vero cells were seeded in 24-well plate at 1×10^5^ cells/well and cultured overnight in DMEM medium containing 10% FBS. The medium was replaced by FBS-free DMEM 30 minutes before infection. Serum samples were heat inactivated at 56°C for 30 minutes and serial diluted to appreciate dilutions in DMEM. Zika virus was diluted to 1×10^3^ PFU/ml in DMEM, mixed with an equal volume of diluted serum samples and incubated at 37°C for 1 hour. After incubation, the mixture was used to infect Vero cells at 37°C for 1 hour. Vero cells were then washed and cultured at 37°C for 96 hours in complete DMEM containing 1% carboxyl methyl cellulose (CMC) and 1.5% FBS. The CMC overlay was carefully removed, the cells were fixed in 4% paraformaldehyde, and stained by crystal violet dye. The percent neutralization was calculated by comparing the plaque numbers of immune sera to that of serum-free control.

### T cell response measurement by ELISPOT

Enzyme-linked immunosorbent spot (ELISPOT) assay was used to detect the IFN-γ secreting murine splenocytes. 96-well PVDF plates (Millipore) were pre-coated with anti-mouse IFN-γ antibody (AN18, Mabtech) at 4°C overnight. Coating antibody was removed and the plate was blocked with RPMI 1640 medium (Gibco) for 1 hour before the addition of the splenocytes. 3×10^5^ freshly isolated mouse splenocytes were added to each well, and then stimulated with 10μg/ml E. coli expressed ZIKV-E80_E, or S2 expressed ZIKV-E80_S; using 10μg/ml DENV-E80 or 10μg/ml HIV polypeptide as antigen specificity controls, 5μg/ml concanavalin A (Con A) as the positive control and PBS as a negative control. The splenocytes were stimulated and cultured at 37°C for 48 hours. After the removal of cells, the plate was incubated with a biotinylated anti-mouse IFN-γ detection antibody (R4-6A2-biotin; Mabtech) diluted in 0.5% FBS PBS (0.2μg/well) for 2 hours, followed by adding 1/1000 diluted alkaline phosphatase (AP)-conjugated streptavidin (Mabtech) for 1 hour. Finally, the plate was colorized by adding TMB substrate (Mabtech) to image the IFN-γ-secreting cells. Spots were counted by a CTL Immunospot reader (Cellular Technology Ltd.).

### Phenotypic and functional analyses by FACS

For flow cytometric (FACS) analysis, 3×10^6^ freshly isolated mouse splenocytes were seeded in 24-well plate and then stimulated with either 10μg/ml E.coli-ZIKV-E80, or 10μg/ml S2-ZIKV-E80, or 10μg/ml DENV-E80, or 10μg/ml HIV peptide, or 50ng/ml PMA and 500ng/ml ionomycin as the positive control, and PBS as a negative control. Golgi plug (BD Biosciences) was added two hours after stimulation at the dilution of 1:1000. After an additional 4 hours, splenocytes were collected and stained with the following antibodies: anti-mouse CD3 (Alexa fluro® 488, Biolegend), anti-mouse CD4 (BV786, BD Bioscience), anti-mouse CD8 (APC, Biolegend), anti-mouse IL-2 (PE, Biolegend), anti-mouse IFN-γ (PE/Cy7, Biolegend), and Aqua fluorescent for live and dead cell discrimination (Life technologies). The stained cells were fixed in 4% paraformaldehyde. FACS analysis was performed on a Fortessa flow cytometer (BD Biosciences), and data were analyzed with FlowJo software (Tree Star).

### Statistical analysis

Antibody ending point titers, PRNT_50_ titers, T cell responses and *in vivo* protection results were evaluated for statistical differences by one-tailed Student’s T test. The curves of antibody titer, neutralizing antibody titer and weight change of infected mice were analyzed by Two-way ANOVA test, followed by Tukey's correction for multiple comparisons. PRNT_50_ were obtained through PROBIT regression analysis (IBM SPSS Statistics). Statistical significance was reported as follows, *p < 0.05; **p < 0.01; ***p < 0.001.

## Results

### Expression of vaccine antigens and immunization strategy

**Fig 1:**
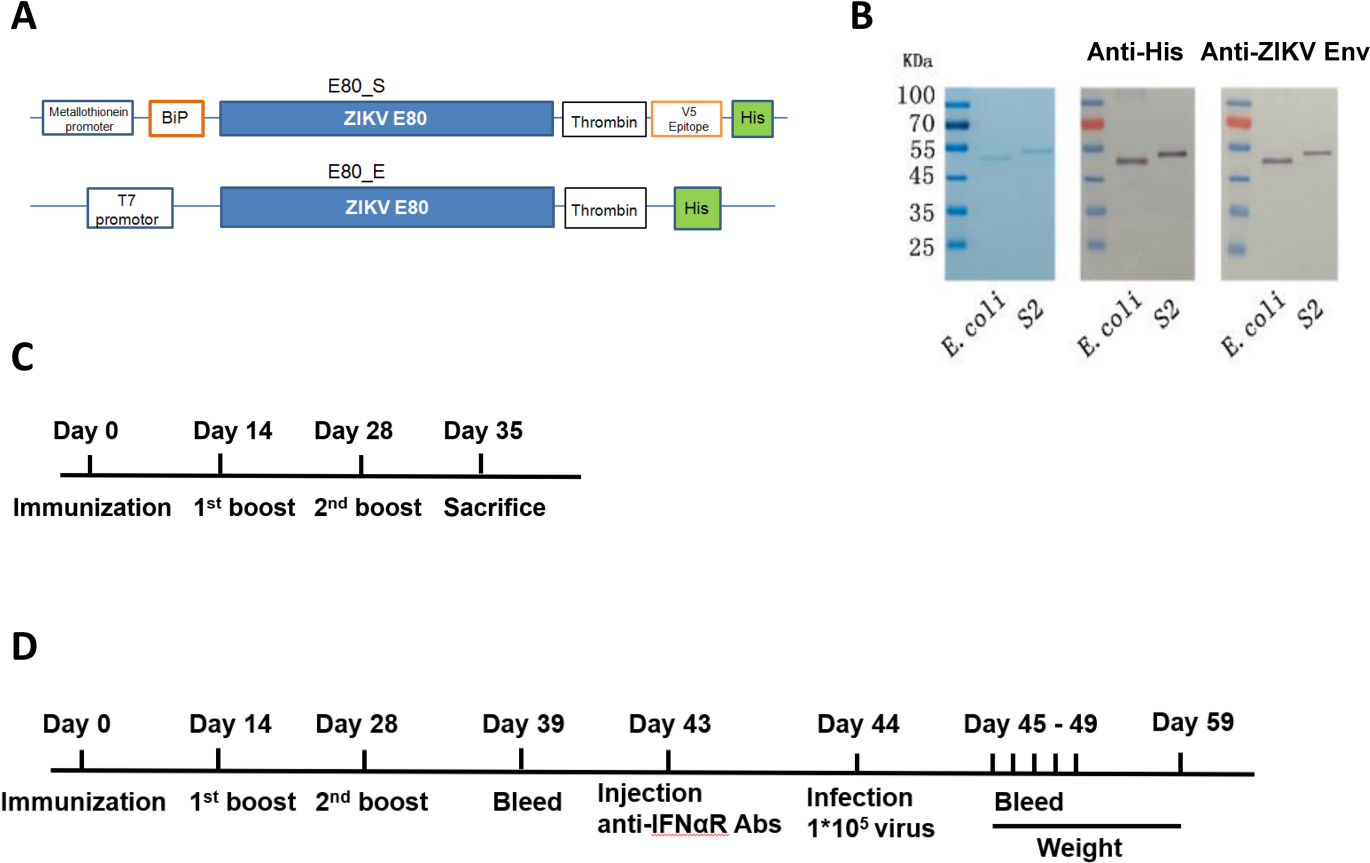
Expression of recombinant ZIKV envelope proteins and immune strategy. (A) Schematics of plasmid constructs expressing E80_S and E80_E. (B) Purified recombinant ZIKV E80 proteins were verified by SDS-PAGE (left panel) and Western Blot with anti-His antibody (middle) or anti ZIKV envelope antibody (right). Experimental strategies: (C) immunization and measurement of cellular immune responses; (D) Immunization and measurement of humoral immune responses and protection against ZIKV challenge.

The envelope protein located on the surface of mature flaviviruses is the principal target for neutralizing antibodies (nAbs) [35]. Thus, we chose to express the ectodomain of ZIKV envelope protein as an immunogen and tested its potential as a vaccine candidate. The sequence of 80% N-terminal of ZIKV envelope protein (E80) was cloned into pET30a and pMT/Bip/V5-His A plasmids for *E*.*coli* (E80_E) and *S2* (E80_S) expression systems, respectively. For further protein purification and detection, a six histidine (6-His) was inserted at the C-terminal of E80 (**Fig 1A**). The expressed proteins were purified by Ni-NTA sepharose beads and verified to be at the correct size of 47-48kDa by SDS-PAGE and Western blot analysis using anti-His or anti-ZIKV E protein antibodies **(Fig 1B**). Protein yields were approximately 10mg/L for E80_E and 18mg/L for E80_S. The purified proteins were stored at 1mg/ml in a -80°C freezer until use.

Mice were immunized subcutaneously with E80_E (10μg or 50μg) or E80_S (10μg or 50μg) emulsified in aluminum (Alhydrogel) or PBS in aluminum as a negative control at weeks 0, 2 and 4. A proportion of the mice in each group were sacrificed for evaluating T cell responses one week after the last immunization **(Fig 1C)**, and the remaining mice were bled for testing antibody responses and vaccine protection upon ZIKV challenges **(Fig 1D)**. In the protection assay, immunized mice were first injected with type I IFN receptor blocking antibody MAR1-5A3, and then inoculated with 1*10^5^ PFU of ZIKV on the next day. The infected mice were bled from 1 to 5 days post infection (DPI) and weighed daily for two weeks after infection (**Fig 1D**).

### ZIKV E80 vaccines elicited humoral immune responses

**Fig 2:**
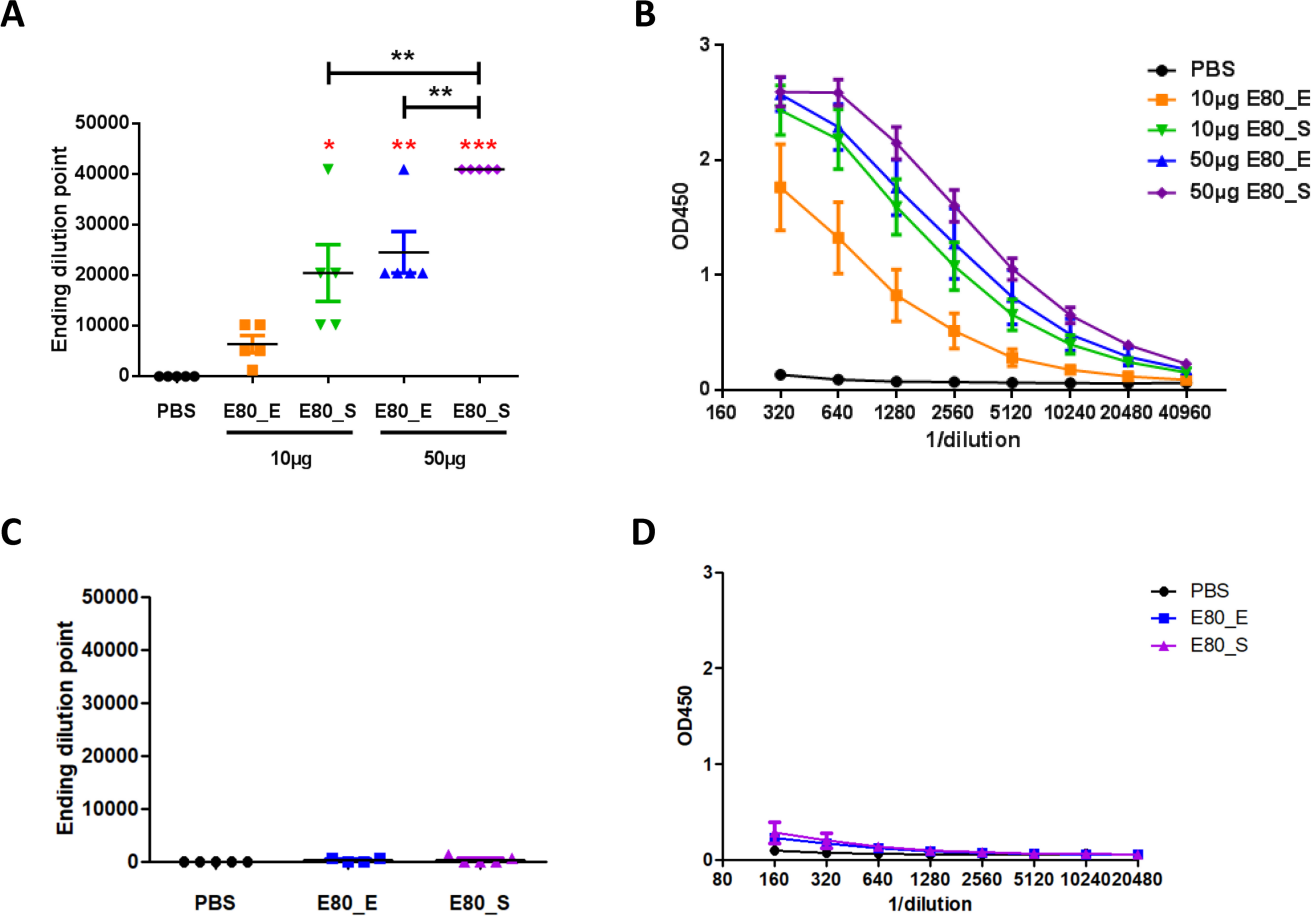
Measurement of ZIKV specific and DENV cross-reactive antibody responses elicited by ZIKV E80_S and E80_E proteins. (A) and (B) Serum samples from different groups of mice (n = 5 for each group) were 2-fold serially diluted from 1:320 to 1:40960 and measured for (A) end-point dilution titers and (B) OD_450_ values by using ZIKV E80 protein coated ELISA. (C) and (D) Serum sample obtained from mice received PBS (n = 5), 50μg E80_E (n = 4) and 50μg E80_S (n = 5) were 2-fold serially diluted from 1:160 to 1:20480 and measured for (C) end-point dilution titers and (D) OD_450_ values by using DENV-3 E80 protein coated ELISA. The statistical differences between PBS control and immunization groups were determined by Student’s t test, and marked with black stars. A p<0.05 value was designated as *, p<0.01 as **, and p<0.001 as ***. Red stars indicate statistical differences between the 10μg E80_E group and other immunization groups. The data were presented as mean ± SEM. (A) and (B) Each sample was assayed in duplicates and the figures are representative results of 4 independent experiments. (C) and (D) Each sample was assayed in duplicates and the figures are representative results of 2 independent experiments.

We first evaluated the humoral immune responses in immunized and control mice by ELISA. Compared to the control group, mice immunized with both forms of E80 proteins elicited high levels of antigen specific binding antibodies. The average end-point dilutions were 1/5,000 to 1/40,000 for the lowest and highest responses, respectively (**Fig 2A**). Increase in antigen dose resulted in the improvement of antibody levels in both cases (**Fig 2B**). For the same dosing groups (10μg or 50μg), E80_S stimulated significantly greater antigen-specific binding antibody responses than E80_E **(Fig 2B)**. Because ZIKV and DENV are two closely related flaviviruses and they share similar viron structure [27], and cross-reactive antibodies may cause antibody dependent enhancement (ADE) between ZIKV and DENV [36], we then tested the cross-reactivity of immune sera from two mice received high dose of ZIKV antigens to DENV-3 E80 protein. Minimal cross-reactivity was detected at a 1:160 dilution of immune sera from 2 mice received E80_E immunization and 3 mice received E80_S immunization (Fig 2C). And there was no significant differences between E80_E immune sera and E80_S immune sera (Fig 2D).

**Fig 3:**
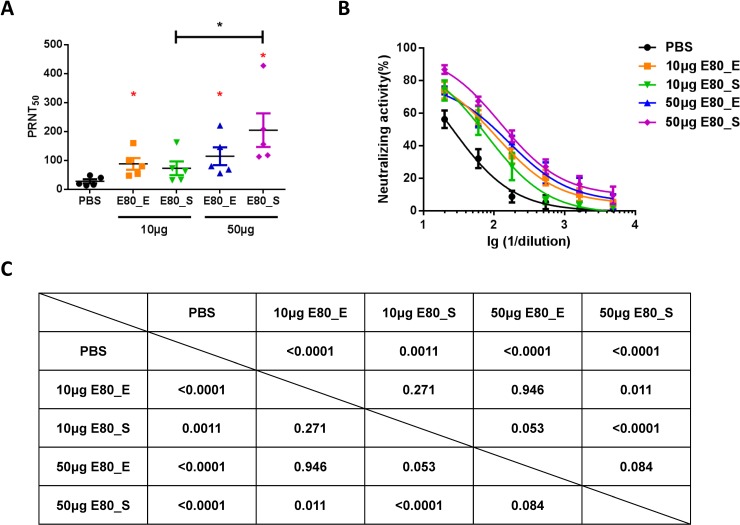
Assessment of serum neutralization activity by plaque reduction neutralization test (PRNT). Serum samples were 3-fold serially diluted from 1:10 to 1:2430, and mixed with an equal volume of medium containing 100 PFU Zika virus then applied on Vero cells. Neutralizing activity was calculated as described in the Materials and Methods. (A) Summary of PRNT_50_ titer of each mouse in different experimental groups. Red stars represent significant differences (Student’s t tests) between the PBS control group and the indicated vaccination group against serum dilutions. (C) Two-way ANOVA test, followed by Tukey's correction for multiple comparisons were used to determine statistical differences among curves plotted in (B). The data were presented as mean ± SEM. Each sample was assayed in duplicates and the figures are representative results of 4 independent experiments.

Having demonstrated that both E80 proteins stimulated antigen specific antibody responses, we next assessed whether these immune sera had neutralization capacity against ZIKV by performing plaque assays. All immunized mice, except for the 10μg E80_S group (P value = 0.067), generated significantly higher levels of neutralizing antibodies than the control group, with the highest PRNT_50_ value 427.3 in the 50μg E80_S group. Mice immunized with 50μg E80_S elicited stronger neutralizing antibody responses than that of the same antigen at a lower dose, whereas no improvement in neutralization potency was observed in the higher dose of E80_E compared to the lower dose **(Fig 3A)**. Data analyses by two-way ANOVA regarding different antigens and dilutions revealed that all immunized groups including 10μg E80_S group displayed significantly stronger neutralization activities, compared with PBS control group **(Fig 3B and 3C)**.

### ZIKV E80 vaccines induced cellular immune responses

**Fig 4:**
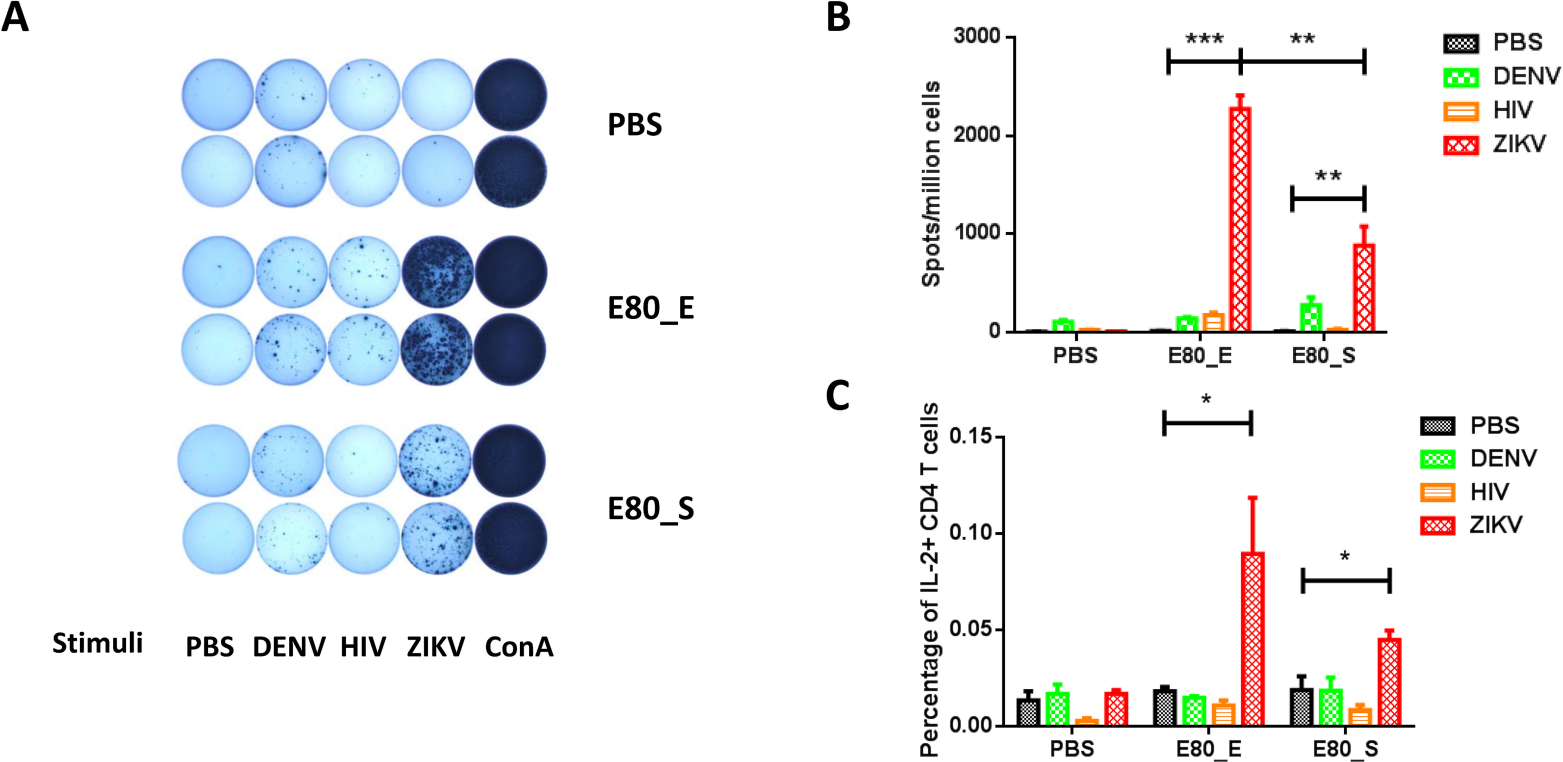
Measurement of antigen specific T cell responses by ELISPOT and ICS assays. (A) Representative ELISPOT results. Splenocyte of PBS, 50μg E80_E and 50μg E80_S immunized mice were stimulated with PBS (lane 1), DENV E80 protein (lane 2), HIV synthesized polypeptide (lane 3), ZIKV E80 protein (lane 4) and Con A (lane 5). (B) Summary of the ELISPOT results. Splenocytes were cultured at 37°C for 48 hours with the same dose of (10μg/ml) ZIKV E protein, DENV E protein, HIV protein or PBS. IFNγ ELISPOT assay was performed to quantify antigen specific T cells at 48 hours after stimulation. (C) Summary of the ICS results. Splenocytes from immunized mice were stimulated with either 10μg/ml of ZIKV E protein, or 10μg/ml of DENV E proteins, or 10μg/ml of HIV protein or PBS for 6 hours. Golgi stop was supplemented 2 hours after stimulation. Statistical differences were analyzed by Student’s t tests. Different p values were indicated by * (p<0.05), or ** (p<0.01), or *** (p<0.001). The data were presented as mean ± SEM. The figures are representative results of 2 independent experiments. In ELISPOT assay, each sample was assayed in duplicates; in ICS assay, each sample was measured once.

Because cellular immune responses are usually desirable and they can help the induction of antibody responses, we also examined T cell activation by ZIKV E80 antigens. Mice were immunized 3 times with either 50μg of each immunogen or PBS control. Splenocytes were harvested for assessment of T cell responses by IFNγ-ELISPOT assay using stimuli ZIKV-E80, DENV-E80, an HIV polypeptide and PBS as negative controls, or Con A as the positive control (**Fig 4A**). For both protein-immunized groups, only ZIKV specific T cell responses were detected, and E80_E induced stronger IFNγ responses than that of E80_S upon ZIKV-E80 stimulation (**Fig 4B**). To further characterize the phenotype of responding T cells, we performed additional cytokine intracellular-staining and FACS analysis. Results showed that CD4+ T cells produced IL-2 only in response to ZIKV-E80 protein stimulation, irrespective of the protein expressed in *E*.*coli* or S2 cells (E80_E or E80_S), but not by simulation with a closely-related antigen (DENV-E80) or an entirely different antigen (HIV polypeptide) (**Fig 4C**). Overall, there were limited cross-reactive cellular responses between ZIKV E protein and DENV E protein (**Fig 4B and 4C**).

### ZIKV E80 vaccines confer protection against viral challenge in immunocompetent mice

**Fig 5:**
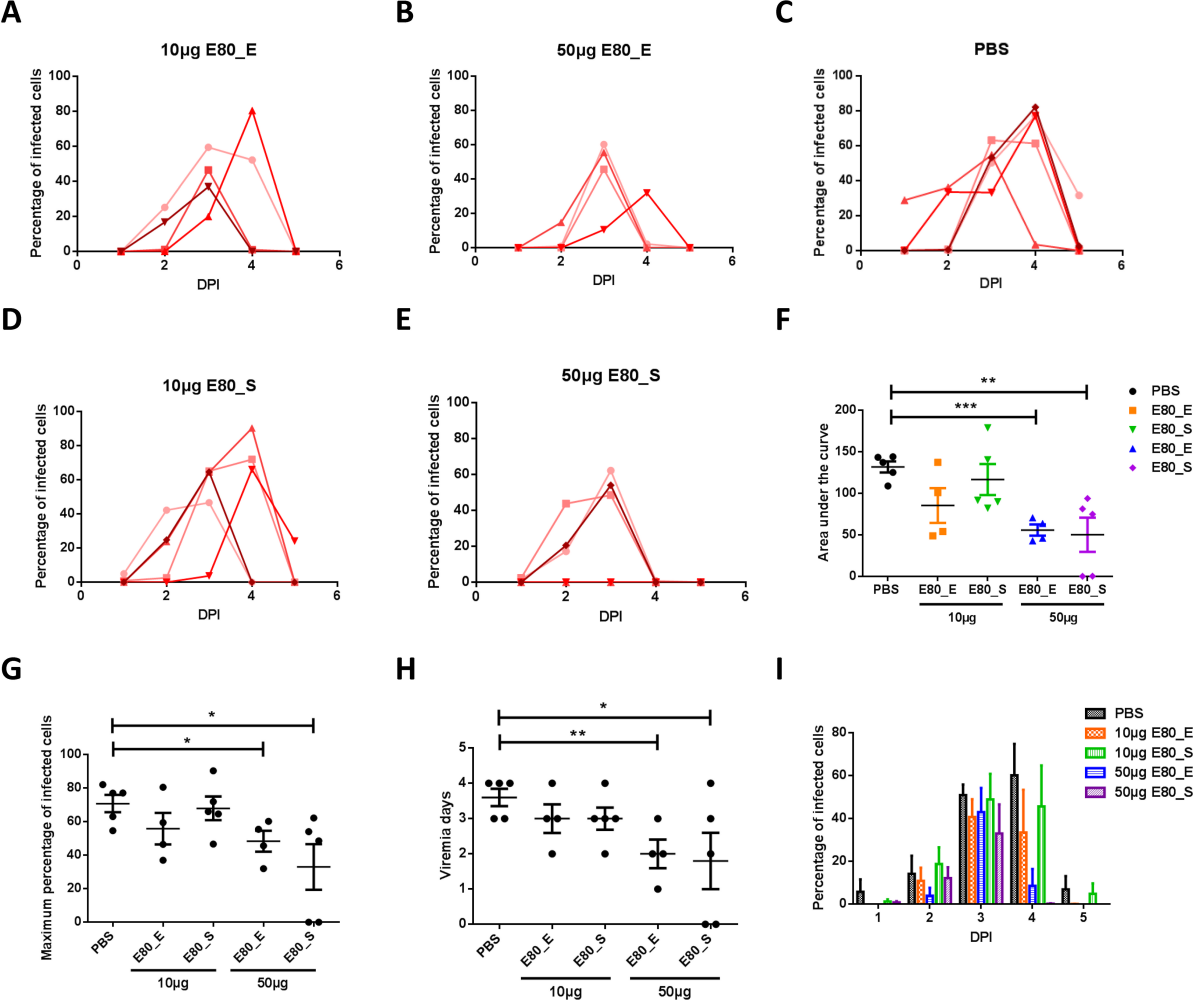
Protective efficacy of vaccines against ZIKV viremia. Viremia levels of immunized mice after ZIKV challenge were presented by percentage of infected Vero cells. 10μl of serum samples obtained at various days post infection were applied to Vero cells in DMEM containing 1% FBS for 4 days. The percentage of infected cells was determined by staining with an anti-E antibody 4G2, followed by an anti-mouse AF488 conjugated antibody, and then analyzed by flow cytometry. (A) 10μg E80_E group (n = 4), (B) 50μg E80_E group (n = 4), (C) PBS group (n = 5), (D) 10μg E80_S group (n = 5), and (E) 50μg E80_S group (n = 5). Each line represented one mouse. (F) Area under the curve (AUC) was calculated by Graphpad Prism 6 software. (G) Maximum percentage of infected cells from 1 to 5 DPI for each experimental group. (H) Duration of detectable viremia. (I) Summary of viremia data for each of the groups at days 1 to 5 post infection. The data were presented as mean ± SEM. Statistical differences were determined by Student’s t test and p values were indicated by * (p<0.05), or ** (p<0.01), or *** (p<0.001). The figures are representative results of 2 independent experiments.

To examine whether immune responses elicited by vaccination can confer protection against ZIKV infection *in vivo*, we used a recently published method [37], with minor modifications. Wild type BALB/C mice were immunized 3 times with either 10 μg or 50 μg ZIKV-E80 proteins or PBS control, and then an anti-type I IFN receptor antibody was administered to each mouse one day prior to infection by ZIKV at 1x10^5^ pfu/mouse (as shown in the schematics in **Fig 1C**). The viremia levels were determined from 1 to 5 DPI via inoculation of 10μl serum samples onto Vero cells for a 4-day incubation and subsequent detection of infected cells by FACS analysis. Results showed that mice immunized with PBS developed 5 days of detectable viremia from 1 day post-infection, reaching as high as 60–80% of infected cells on day 3 or 4 after challenge (**Fig 5C**). For protein immune groups, mice immunized with 10μg of either E80_E or E80_S exhibited similar patterns of viremia as compared with PBS control group (**Fig 5A and 5D**), while immunization with 50μg of the antigens afforded partial protection by shortening the duration of viremia and reducing the magnitude of peak infection (**Fig 5B and 5E**). Notably, 2/5 mice received 50μg E80_S antigen were completely protected from ZIKV infection as demonstrated by undetectable infectious viruses in sera (**Fig 5E and 5F**).

To more carefully analyze the viral dynamic patterns, we compared results from ZIKV challenge experiments among these groups using different readouts. We first computed area-under-the-curve (AUC) for each experimental group, and discovered that the average AUC of mice received high dose (50μg) of antigens were significantly smaller than PBS controls (p<0.01) (**Fig 5F**). Likewise, the maximal number of infected cells was significantly lower in the 50μg E80_E and 50μg E80_S groups (**Fig 5G**); and the duration of viremia was shorter in the high dose groups (**Fig 5H**). On average, the percentage of infected cells were the lowest for mice that received 50μg E80_E on days 2 and 4 among all experimental groups; those received 50μg E80_S also had fewer infected cells on day 4 post infection, while by day 5, the number of infected cells returned to the baseline level which was comparable to day 1 (**Fig 5I**).

**Fig 6:**
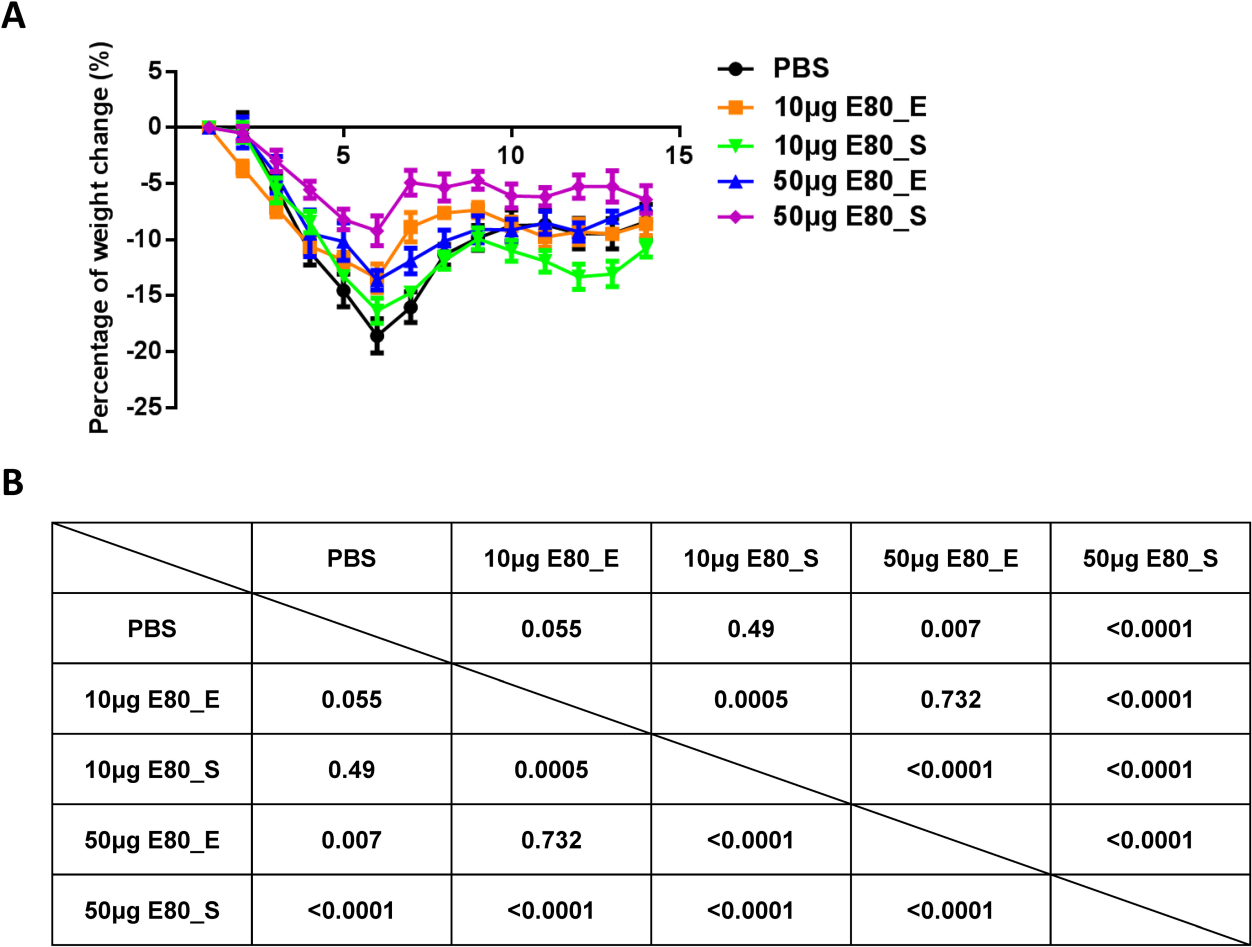
Weight changes of mice in each experimental group. (A) Summary of weight changes of immunized mice upon ZIKV infection from day 1 to 14 DPI. (B) Two-way ANOVA test was performed on data in (A), followed by Tukey's correction for multiple comparisons. The data were presented as mean ± SEM. The figures are representative results of 2 independent experiments.

Many viral infections lead to weight loss of infected mice [17, 38]. Thus, another indicator of protection against viral infection is preventing weight loss. To examine the protective efficacy of ZIKV E80 protein based vaccine, the weight of mice was measured daily for 14 days post infection. Results showed that all vaccine groups, except for low dose of E80_E, had significantly less weight loss than the PBS control group (p<0.0005 or p<0.0001) as determined by one-way ANOVA analysis (**Fig 6**). Of note, mice received 50μg E80_S had the least weight loss post infection.

**Fig 7:**
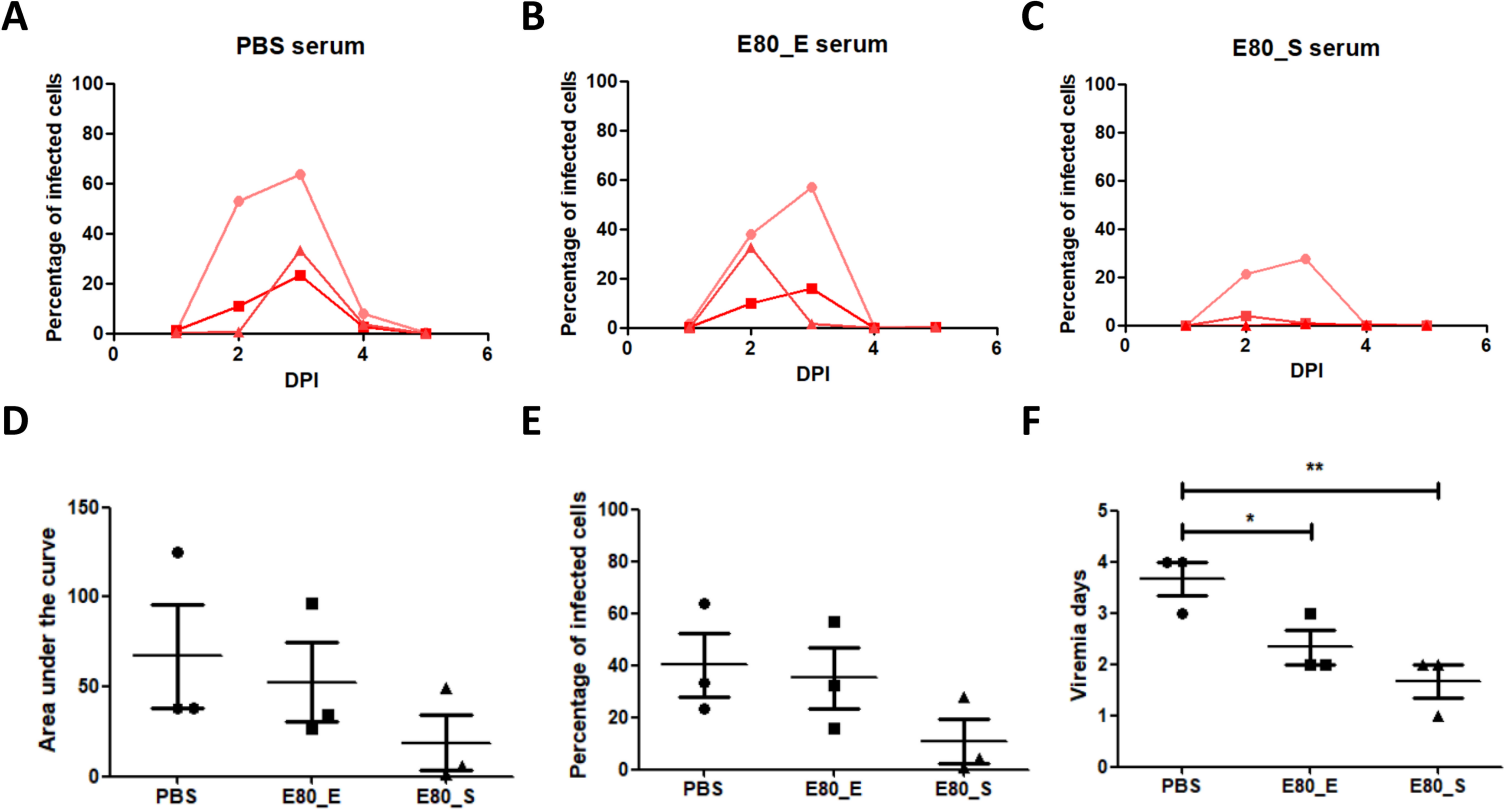
Assessment of protection by adoptively transfer of ZIKV immune sera. Each mouse received 300μl of pooled sera from either control (A) or 50μg ZIKV E80 immune (B, C) mice and then challenged. Viremia level of each group of recipient mice (n = 3) was measured and presented as percentage of infected cells. Each line represented one mouse. (D) AUC was calculated by using the Graphpad Prism 6 software. (E) Maximum percentage of infected cells from 1 to 5 DPI was measured for each experimental group. (F) Duration of detectable viremia. The data were presented as mean ± SEM. Statistical differences were determined by Student’s t test. The p values were indicated by * (p<0.05), or ** (p<0.01), or *** (p<0.001).

To further determine whether antibody responses could confer protection independently, we adoptively transferred sera from control and immunized mice to naïve mice, which were then experimentally infected by ZIKV. The viremia levels in mice were recorded from 1 DPI to 5 DPI **(Fig 7A**–**7C)**. Compared to the PBS group, 50μg E80_E group did not display significant differences in either AUC or peak of viremia **(Fig 7D and 7E)**. However, two of three mice received sera from 50μg E80_S immunized mice showed dramatically reduced viremia level **(Fig 7C)**. Both groups of immunized mice showed significantly reduced duration of infection **(Fig 7F)**.

## Discussion

To explore a new strategy for developing a Zika virus vaccine, we tested two forms of ZIKV E80 protein produced by prokaryotic expression system (*E*. *coli*) and eukaryotic (S2) expression systems. In immune competent mice, both E80 proteins elicited robust binding and neutralizing antibody responses after three immunizations and protected mice against experimental ZIKV challenge. The antibodies elicited by ZIKV E80 antigens showed minimal cross-reactivity to DENV E80 protein. In contrast to many previous Zika vaccine studies that examined vaccine immunogenicity in normal mice, but protective efficacy in immune compromised mice [16, 39, 40], the current experimental system allows for investigation of both immunogenicity and protection conferred by a candidate flavivirus vaccine in the same animals.

It is well documented that DENV and ZIKV do not readily establish infection in immune competent mice. However, by employing a recently developed model that transiently suppress type I interferon pathway by the administration of IFNαR antibody *in vivo* [37], we showed that a short window of 4–5 days of viremia could be detected, and such a window is sufficient for investigation of vaccine-induced protection against viral challenge. Despite some have suggested that normal immune responses could be generated in mice defective in the type I IFN pathway[39], the general consensus is that an intact type I interferon response is essential for priming and maintenance of long-term memory [41–43], both of which are key elements of immunization. Therefore, we suggest that immune competent animal is a better choice for the examination of the full range of immune responses elicited by a vaccine.

Two prokaryotic expressed ZIKV EDIII and E90 protein vaccines have been reported to elicit protective immunity in mice [23, 44]. The dogma is that eukaryotic expression system is preferred than prokaryotic expression system to express protein immunogens that designed to elicit neutralizing antibodies, but formal comparison in the same experimental system are rare. Thus, we sought to compare the eukaryotic expressed E80_S with prokaryotic expressed E80_E side by side. Unexpected, in this study, we did not find marked differences in immunogenicity and protective efficacy between the ZIKV E80 proteins expressed by prokaryotic and eukaryotic systems. In principal, protein expressed in S2 system can be folded and glycosylated properly and assembled more close to the native conformation on virion [45]. For a closely related virus, DENV, there are two glycosylation sites, Asn67 and Asn153. Glycosylation at Asn67 of DENV envelope protein was important for viral attachment to several cell types, and blocking of which reduced viral infectivity [46, 47]. In ZIKV, there is only one glycosylation site at Asn154[26], the functional importance of which is not yet clear. However, replacing ZIKV glycan loop by DENV glycan loop could increase the sensitivity to neutralizing Abs which targeted EDII fusion loop[48]. And the residues surrounding the ZIKV E protein glycan regulated virus antigenicity independently of the presence of a glycan[48]. Whether the induction of glycoprotein specific antibodies plays a similar role for the protection against ZIKV as it did for DENV warrants further study. Another possible explanation is that protection against ZIKV infection is mediated by several arms of immune responses including T cell responses. Therefore, a minor imperfection of the vaccine antigen at eliciting antibody response is compensated by its ability to activate other type of protective immune responses. Proteins expressed using *E*.*coli* system lack glycosylation, but can still be used for certain vaccines. For instance, *E*.*coli* expressed hepatitis E virus (HEV) virus-like-particle (VLP) had been shown to confer protection against HEV in human [49]. There were also reports that DENV envelope protein produced in *E*.*coli* could protect against lethal DENV infection in mice [50, 51].

Which domains of the ZIKV envelop protein are the most optimal antigen for formulating an efficacious Zika vaccine is incompletely understood. At least in human subjects infected by ZIKV, monoclonal antibodies to all three E domains have been isolated and showed to potently neutralize ZIKV [52]. However, there has also reported that EDIII targeted antibody could efficiently protect mice from ZIKV infection [38, 53]. For other flaviviruses, such as DENV and WNV, antibody targeted EDIII potently neutralized virus infection [54–56]. However, there was only 5% ZIKV EDIII specific B cells among all B cells recognizing ZIKV envelope protein in infected patients [53]. There were also some antibodies that targeted EDI-EDII epitopes having high neutralization activity [28, 53]. Additionally, some EDIII targeted antibodies could not neutralize all strains of ZIKV [53]. Thus, the pros and cons of EDIII versus E80 as vaccine immunogens await further investigation, preferably by a head-to-head study in the same experimental settings.

The relative contribution of humoral and cellular immune responses to vaccine inducted protection is not entirely clear. In one study, the removal of T cells had little impact on DNA vaccine conferred protection against ZIKV challenge [57]. In other studies, CD8 deficient mice displayed higher mortality after ZIKV infection [29, 57]. In our study, E80_S and E80_E both evoked potent cellular and humoral immune responses.

Despite having elicited desirable immune responses in mice, we should not directly translate these results into humans. The fact is that vaccine-elicited human and murine immune responses might be distinct. For example, in DENV, a flavivirus closely related to ZIKV, vaccine responses in human clinical trials were different from those observed in pre-clinical studies. Chimeric YF/DEN-2 vaccine showed great immunogenicity in mice and non-human primates [58–60], but the protective efficacy to DENV-2 was minimal in human subjects received a tetravalent comprised of the chimeric YF/DEN-2 vaccine and three other chimeric dengue vaccines to the other 3 DENV serotypes, as demonstrated in both phase 2b and phase 3 clinical trials[61, 62]. Thus, whether the observed small differences in immunogenicity and protective efficacy between E80_S and E80_E in mice will have any impact in human studies are uncertain. An intermediate experiment using non-human primate may help to clarify this issue.

In summary, we have constructed recombinant protein based ZIKV vaccines and demonstrated their protective efficacy in immunocompetent mice. This work adds another candidate ZIKV vaccine to the existing vaccine modalities including DNA vaccine, RNA vaccine, purified inactivated virus (PIV) vaccine, live attenuated virus vaccine, and Adenovirus vectored vaccine [16–21, 39, 63]. Simultaneously development of multiple vaccine candidates, we believe, will facilitate the rapid marketing of one or several human vaccines, and contribute to the prevention of ZIKV infection globally.

## Supporting information

S1 Table Original data for Fig 2. (PDF)

S2 Table Original data for Fig 3. (PDF)

S3 Table Original data for Fig 4. (PDF)

S4 Table Original data for Fig 5. (PDF)

S5 Table Original data for Fig 6. (PDF)

S6 Table Original data for Fig 7. (PDF)
