# Supplementary material for: Recombinant Zika virus envelope protein elicited protective immunity against Zika virus in immunocompetent mice
Source: PLoS One. 2018 Mar 28;13(3):e0194860. doi: 10.1371/journal.pone.0194860 (PMC5874044; doi:10.1371/journal.pone.0194860)
Supplement: S1 Table — (PDF) [file pone.0194860.s001.pdf]

S1 Table. Original data for Fig 2

Fig 2A Ending point dilution

	PBS	10μg E80_E	10μg E80_S	50μg E80_E	50μg E80_S
mouse 1	1	5120	10240	20480	40960
mouse 2	1	5120	20480	20480	40960
mouse 3	1	10240	20480	20480	40960
mouse 4	1	10240	40960	20480	40960
mouse 5	1	1280	10240	40960	40960

Fig 2B OD450

1/dilution		PBS			
	mouse number	1	2	3	4
320		0.109392	0.071933	0.150039	0.206017
640		0.07871	0.067127	0.099779	0.118508
1280		0.073349	0.070083	0.074906	0.080409
2560		0.084233	0.060585	0.067437	0.069036
5120		0.065056	0.061123	0.061106	0.068451
10240		0.06392	0.05956	0.05768	0.058801
20480		0.061195	0.061713	0.057123	0.057934
40960		0.065025	0.067989	0.058291	0.059302

1/dilution	10μg E_E80				
	1	2	3	4	5
320	1.33375	2.55708	0.509114	2.270295	2.155565
640	1.032719	2.183985	0.345491	1.709775	1.35698
1280	0.615261	1.539725	0.216192	1.079065	0.678176
2560	0.335188	0.946502	0.13449	0.794635	0.361582
5120	0.22392	0.515502	0.095304	0.369262	0.207046
10240	0.147691	0.308367	0.077056	0.214876	0.134022
20480	0.105905	0.186775	0.065492	0.136234	0.093775
40960	0.093065	0.118715	0.061687	0.095057	0.078541

1/dilution	50μg E_E80					10μg S_E80					50μg S_E80				
	1	2	3	4	5	1	2	3	4	5	1	2	3	4	5
320	2.469765	2.219875	2.64947	2.438755	3.100225	1.850675	2.51116	2.61679	3.0994	2.103445	2.201535	2.47022	2.968565	2.608495	2.737485
640	2.01703	1.71548	2.261515	2.71091	2.74234	1.426025	2.649315	2.27295	2.79767	1.764165	2.74631	2.648265	2.70826	2.13802	2.706005
1280	1.43718	1.195365	1.64979	2.57683	1.96337	0.954428	2.26354	1.537175	2.00263	1.221095	2.57339	2.185715	1.971575	1.746945	2.273205
2560	0.942043	0.75054	0.967103	2.45627	1.262945	0.613959	1.74464	0.959434	1.339955	0.738593	2.11529	1.54285	1.335335	1.38552	1.64867
5120	0.569199	0.463845	0.55114	1.73605	0.727266	0.36574	1.08719	0.566097	0.823866	0.433372	1.388435	0.948853	0.841043	0.988554	1.104615
10240	0.352687	0.298513	0.324523	1.03713	0.406577	0.231489	0.668545	0.359929	0.476497	0.253922	0.875072	0.552802	0.473557	0.673086	0.684155
20480	0.216265	0.203769	0.201279	0.593509	0.239868	0.151773	0.385893	0.243752	0.282189	0.154701	0.513544	0.326315	0.302987	0.43517	0.381723
40960	0.150903	0.1442	0.129534	0.325527	0.145827	0.115443	0.230613	0.148578	0.172756	0.098364	0.2797	0.203009	0.180502	0.259651	0.216417

Fig 2C Ending point dilution

	PBS	50μg E80_E	50μg E80_S
mouse 1	1	1	1
mouse 2	1	640	1
mouse 3	1	1	1
mouse 4	1	640	640
mouse 5	1		1280

Fig 2D OD450

1/dilution		PBS					50μg E80_E				50μg E80_S				
	mouse number	1	2	3	4	5	1	2	3	4	1	2	3	4	5
160		0.130193	0.082676	0.084215	0.086999	0.10922	0.270982	0.211258	0.326215	0.127033	0.130873	0.316562	0.703887	0.107093	0.167144
320		0.085565	0.066929	0.06935	0.07019	0.084451	0.237796	0.14563	0.219033	0.092523	0.097957	0.225303	0.494915	0.081529	0.119559
640		0.071408	0.061066	0.06217	0.062377	0.072484	0.168948	0.105603	0.142548	0.086143	0.07855	0.149344	0.310531	0.069387	0.093614
1280		0.062418	0.058298	0.058551	0.059171	0.065499	0.108281	0.078239	0.098351	0.06826	0.066606	0.109008	0.191504	0.063505	0.077747
2560		0.0628	0.057853	0.055709	0.056936	0.061131	0.081121	0.068442	0.075436	0.064447	0.059572	0.080293	0.127925	0.060881	0.066241
5120		0.056674	0.056372	0.065561	0.062088	0.059518	0.065897	0.062021	0.064255	0.061932	0.057079	0.066402	0.09206	0.059749	0.06317
10240		0.055786	0.058901	0.054342	0.086758	0.059979	0.059941	0.059074	0.059151	0.060429	0.056521	0.06017	0.073908	0.060423	0.062253
20480		0.057855	0.058766	0.054424	0.054097	0.058838	0.057504	0.058194	0.057844	0.06055	0.056024	0.057535	0.06545	0.059138	0.063086

0
