# Supplementary material for: Recombinant Zika virus envelope protein elicited protective immunity against Zika virus in immunocompetent mice
Source: PLoS One. 2018 Mar 28;13(3):e0194860. doi: 10.1371/journal.pone.0194860 (PMC5874044; doi:10.1371/journal.pone.0194860)
Supplement: S2 Table — (PDF) [file pone.0194860.s002.pdf]

S2 Table. Original data for Fig 3

Fig 3A	PRNT50					
		PBS	10μg E80_E	10μg E80_S	50μg E80_E	50μg E80_S
	mouse 1	15.9	47.4	33.2	80.3	113
	mouse 2	19.3	99.6	63.2	55.6	118.3
	mouse 3	40.4	53.4	72.7	68.6	156
	mouse 4	13.6	79.6	31.6	220.8	208.2
	mouse 5	48.4	159.6	162.1	146.5	427.3

Fig 3B	Neutralizing activity (%)						
	lg(1/dilution)		PBS				
		mouse number	1	2	3	4	5
	1.30103		49.68554	54.08805	70.90909	41.04478	65.48672
	1.778151		19.49685	22.64151	43.18182	26.1194	48.67257
	2.255272		1	1	7.727273	15.29851	18.58407
	2.732394		1	1	1	1	22.12389
	3.209515		1	1	1	1	1
	3.686636		1	1	1	1	1

lg(1/dilution)	10ug E80_E					50ug E80_E				
	1	2	3	4	5	1	2	3	4	5
1.30103	62.71357	90.35533	63.44538	77.70035	76	74.86339	64.85149	78.33334	76.1745	75
1.778151	55.7789	62.43655	50.42017	48.08362	71.2	50.81967	48.0198	50.41667	61.4094	59.25926
2.255272	23.61809	27.91878	32.35294	33.79791	50.4	48.63388	31.68317	34.16667	47.65101	49.07407
2.732394	14.57286	15.22843	14.70588	18.11847	39.2	12.56831	22.27723	5.833333	36.57718	38.88889
3.209515	16.58291	1	1	19.86063	11.2	1	2.970297	1	35.90604	24.07407
3.686636	11.55779	1	1	7.665505	1	1	1	1	23.8255	1

lg(1/dilution)	10ug E80_S					50ug E80_S				
	1	2	3	4	5	1	2	3	4	5
1.30103	58.85714	71.78218	86.76471	62.64151	89.24731	82.29166	86.72566	94.90196	79.87805	89.74359
1.778151	46.28571	50	61.39706	33.58491	86.02151	68.75	60.61947	66.27451	64.32927	76.92308
2.255272	8.571428	33.16832	18.01471	19.24528	56.98925	39.58333	38.49557	43.13726	49.39024	58.11966
2.732394	1	10.39604	1	9.433962	9.67742	18.75	19.9115	21.17647	36.28049	39.31624
3.209515	1	11.38614	1	1.886792	1	3.125	11.50443	11.76471	24.69512	30.76923
3.686636	1	4.455446	1	1.886792	1	1	1	1	19.20732	25.64103
