# Supplementary material for: Recombinant Zika virus envelope protein elicited protective immunity against Zika virus in immunocompetent mice
Source: PLoS One. 2018 Mar 28;13(3):e0194860. doi: 10.1371/journal.pone.0194860 (PMC5874044; doi:10.1371/journal.pone.0194860)
Supplement: S3 Table — (PDF) [file pone.0194860.s003.pdf]

S3 Table. Original data for Fig 4

Fig 4B	Spots/million cells																
		stimulation	PBS				DENV				HIV				ZIKV		
		mouse number	1	2	3		1	2	3		1	2	3		1	2	3
	PBS		3	10	5		112	75	132		13	32	23		8	8	8
	E80_E		10	12	17		127	173	118		147	150	227		2040	2510	2267
	E80_S		13	5	5		420	148	262		42	12	20		968	1172	508

Fig 4C	Percentage of IL-2+ CD4 T cells (%)																
		stimulation	PBS				DENV				HIV				ZIKV		
		mouse number	1	2	3		1	2	3		1	2	3		1	2	3
	PBS		0.017	0.004	0.019		0.007	0.021	0.022		0.004	0	0.004		0.017	0.013	0.02
	E80_E		0.014	0.022	0.018		0.015	0.013	0.016		0.006	0.015	0.011		0.051	0.14	0.089
	E80_S		0.012	0.033	0.011		0.011	0.032	0.012		0.013	0.008	0.004		0.048	0.051	0.035
