# Supplementary material for: Recombinant Zika virus envelope protein elicited protective immunity against Zika virus in immunocompetent mice
Source: PLoS One. 2018 Mar 28;13(3):e0194860. doi: 10.1371/journal.pone.0194860 (PMC5874044; doi:10.1371/journal.pone.0194860)
Supplement: S4 Table — (PDF) [file pone.0194860.s004.pdf]

S4 Table. Original data for Fig 3

Fig 5A	Percentage of infected cells (%)					
	DPI	mouse number	1	2	3	4
	1		0.018	0.016	0.045	0.021
	2		25.3	1.23	0.11	16.9
	3		59.5	46.5	20	37
	4		52.3	1.05	80.5	0.056
	5		0.071	0.078	0.25	0.044

Fig 5B	Percentage of infected cells (%)					
	DPI	mouse number	1	2	3	4
	1		0.027	0.068	0.028	0.013
	2		0.92	0.082	14.9	0.049
	3		60.3	45.7	55.5	10.6
	4		2.31	0.05	0.021	32
	5		0.046	0.038	0.027	0.045

Fig 5C	Percentage of infected cells (%)						
	DPI	mouse number	1	2	3	4	5
	1		0.029	0.02	28.9	0.026	0.022
	2		0.092	0.7	36.1	33.5	0.45
	3		50.3	63.1	54.7	33.3	53.2
	4		77	61.3	3.46	77.1	82.1
	5		31.6	0.049	0.054	0.065	2.63

Fig 5D	Percentage of infected cells (%)						
	DPI	mouse number	1	2	3	4	5
	1		5.05	0.98	0.04	0.068	0.023
	2		42.3	2.6	23.9	0.031	24.9
	3		46.7	65.2	64.5	3.75	64.7
	4		0.071	72	90.4	66.1	0.049
	5		0.023	0.021	0.018	24.2	0.019

Fig 5E	Percentage of infected cells (%)						
	DPI	mouse number	1	2	3	4	5
	1		1.69	2.47	0.019	0.027	0.029
	2		17.3	43.9	0.025	0.042	20.6
	3		62.3	48.6	0.039	0.041	54.1
	4		0.85	0.048	0.031	0.053	0.04
	5		0.045	0.071	0.047	0.056	0.064

Fig 5F	AUC					
		PBS	10μg E80_E	10μg E80_S	50μg E80_E	50μg E80_S
	mouse 1	143.21	137.14	91.61	63.57	81.32
	mouse 2	125.13	48.827	140.3	45.89	93.82
	mouse 3	108.74	100.76	178.8	70.45	0.128
	mouse 4	143.95	53.989	82.02	42.68	0.1775
	mouse 5	137.08		89.67		74.79

Fig 5G	Percentage of infected cells (%)					
		PBS	10μg E80_E	10μg E80_S	50μg E80_E	50μg E80_S
	mouse 1	77	59.5	46.7	60.3	62.3
	mouse 2	63.1	46.5	72	45.7	48.6
	mouse 3	54.7	80.5	90.4	55.5	0.047
	mouse 4	77.1	37	66.1	32	0.056
	mouse 5	82.1		64.7		54.1

Fig 5H	Viremia days					
		PBS	10μg E80_E	10μg E80_S	50μg E80_E	50μg E80_S
	mouse 1	4	3	3	3	4
	mouse 2	3	3	4	1	3
	mouse 3	4	4	3	2	0
	mouse 4	3	2	3	2	0
	mouse 5	4		2		2
