# Supplementary material for: Recombinant Zika virus envelope protein elicited protective immunity against Zika virus in immunocompetent mice
Source: PLoS One. 2018 Mar 28;13(3):e0194860. doi: 10.1371/journal.pone.0194860 (PMC5874044; doi:10.1371/journal.pone.0194860)
Supplement: S6 Table — (PDF) [file pone.0194860.s006.pdf]

S6 Table. Original data for Fig 7

Fig 7A	Percentage of infected cells (%)				
	DPI	mouse number	1	2	3
	1		0.79	1.23	0.2
	2		52.9	11.1	0.8
	3		63.7	23.4	33.4
	4		8.05	2.51	3.73
	5		0.17	0.16	0.16

Fig 7B	Percentage of infected cells (%)				
	DPI	mouse number	1	2	3
	1		1.53	0.26	0.094
	2		38.1	10.1	32.5
	3		57	16	1.72
	4		0.18	0.13	0.092
	5		0.21	0.19	0.24

Fig 7C	Percentage of infected cells (%)				
	DPI	mouse number	1	2	3
	1		0.11	0.12	0.16
	2		21.2	4.14	0.13
	3		27.7	0.97	0.79
	4		0.12	0.13	0.27
	5		0.19	0.15	0.13

Fig 7D	AUC			
		PBS	E80_E	E80_S
	mouse 1	125.1	96.15	49.17
	mouse 2	37.71	26.46	5.375
	mouse 3	38.11	34.48	1.335

Fig 7E	Percentage of infected cells (%)			
		PBS	E80_E	E80_S
	mouse 1	63.7	57	27.7
	mouse 2	23.4	16	4.14
	mouse 3	33.4	32.5	0.79

Fig 7F	Viremia days			
		PBS	E80_E	E80_S
	mouse 1	4	3	2
	mouse 2	4	2	2
	mouse 3	3	2	1
