# Supplementary material for: Recombinant Zika virus envelope protein elicited protective immunity against Zika virus in immunocompetent mice
Source: PLoS One. 2018 Mar 28;13(3):e0194860. doi: 10.1371/journal.pone.0194860 (PMC5874044; doi:10.1371/journal.pone.0194860)
Supplement: S5 Table — (PDF) [file pone.0194860.s005.pdf]

S5 Table. Original data for Fig 6

Fig 6A	Weight change (g)										
	DPI		PBS					10ug E80_E			
		mouse number	1	2	3	4	5	1	2	3	4
	1		0	0	0	0	0	0	0	0	0
	2		-0.718735	3.632076	0	-4.263758	1.53417	-4.952216	-3.131438	-4.460303	-2.031316
	3		-4.264494	-2.783019	-5.373276	-10.21319	-4.277081	-8.07993	-5.933251	-8.876003	-5.628438
	4		-10.3977	-7.59434	-11.65002	-14.67526	-11.29707	-10.77324	-8.899877	-12.44425	-10.11426
	5		-15.04552	-9.198113	-15.16881	-18.2945	-14.92329	-10.03475	-11.66049	-13.2917	-12.31485
	6		-19.50168	-12.92453	-19.73371	-22.16163	-18.596	-10.64292	-12.56696	-16.72614	-13.83834
	7		-15.38093	-10.99057	-17.73657	-18.59197	-17.43375	-6.950478	-7.622579	-12.75647	-8.167583
	8		-10.54145	-8.773585	-14.45554	-11.1056	-11.80846	-8.03649	-7.045735	-7.805531	-7.575116
	9		-7.85817	-6.556604	-11.65002	-11.15518	-11.99442	-7.732407	-6.139266	-6.378234	-9.098603
	10		-4.98323	-5.896226	-11.45982	-9.816559	-11.52952	-8.688097	-7.086938	-8.608385	-9.987305
	11		-4.98323	-6.556604	-11.69757	-9.023302	-11.11111	-9.77411	-7.540173	-9.634255	-12.10326
	12		-6.468615	-6.792453	-13.21921	-8.824987	-11.94793	-10.7298	-5.933251	-9.85727	-10.66441
	13		-6.516531	-6.698113	-13.59962	-9.41993	-11.11111	-9.339705	-7.993408	-10.79393	-9.818027
	14		-5.893627	-4.009434	-12.69615	-9.618245	-9.669921	-6.429192	-7.704986	-8.697592	-11.46847

DPI	50ug E80_E				10ug E80_S			
	1	2	3	4	1	2	3	4
1	0	0	0	0	0	0	0	0
2	1.359649	-4.504505	0.465549	0.925926	1.724881	0.133393	-1.194539	-2.389937
3	-1.535088	-8.378378	-1.769088	-5.165692	-3.363519	-4.357492	-6.271331	-8.427673
4	-5	-15	-8.054004	-9.649123	-7.158258	-7.381058	-7.892491	-11.2369
5	-5.964912	-14.0991	-10.70763	-9.844054	-12.89349	-12.40551	-14.37713	-13.41719
6	-11.49123	-15.40541	-14.6648	-12.91423	-14.01466	-15.38461	-16.59556	-19.32914
7	-9.649123	-14.63964	-12.80261	-10.47758	-14.10091	-14.13962	-15.40102	-15.30398
8	-8.070175	-12.79279	-10.28864	-9.356725	-9.616214	-12.18319	-13.05461	-12.57862
9	-7.236842	-12.56757	-7.821229	-8.625731	-7.20138	-9.915518	-11.04949	-11.44654
10	-8.596491	-11.93694	-7.402235	-8.576998	-8.408797	-10.89373	-11.94539	-12.66247
11	-7.982456	-11.48649	-6.79702	-7.699805	-9.271237	-11.69409	-13.13993	-13.58491
12	-8.684211	-11.62162	-7.774674	-9.11306	-10.43553	-12.6723	-15.3157	-14.84277
13	-7.938597	-9.72973	-7.216015	-7.261209	-9.874946	-13.07248	-14.24915	-15.05241
14	-7.017544	-7.837838	-6.098696	-6.530214	-8.926261	-11.02712	-10.75085	-12.49476

DPI	50ug E80_S				
	1	2	3	4	5
1	0	0	0	0	0
2	0.178571	-1.315789	1.15942	-2.556054	-0.091075
3	-0.714286	-6.170599	-1.690821	-4.035874	-2.322404
4	-3.973214	-8.121597	-5.120773	-4.26009	-6.193079
5	-5.758929	-11.29764	-7.05314	-8.744394	-8.105647
6	-8.125	-14.15608	-6.376812	-7.757847	-9.608378
7	-3.839286	-8.711433	-2.125604	-4.304933	-5.555555
8	-4.866071	-9.845735	-2.753623	-5.201794	-4.007286
9	-4.241071	-7.667877	-4.68599	-3.228699	-3.688524
10	-5	-9.664247	-6.859903	-5.650224	-3.324226
11	-4.821429	-9.165154	-6.280193	-5.470852	-5.054645
12	-3.883929	-8.802177	-6.038647	-2.825112	-4.735883
13	-2.1875	-9.528131	-6.618358	-2.331839	-5.601093
14	-5.580357	-10.75318	-6.908213	-3.408072	-5.373406
